# Supplementary material for: CRISPR-dCas13-tracing reveals transcriptional memory and limited mRNA export in developing zebrafish embryos
Source: Genome Biol. 2023 Jan 19;24:15. doi: 10.1186/s13059-023-02848-6 (PMC9854193; doi:10.1186/s13059-023-02848-6)
Supplement: Supplementary file 1 — Additional file 1: Fig. S1. dCas13 protein purification and assembly with gRNA. Fig. S2. Screening the capable dCas13 proteins to label 48× GCN4 RNA. Fig. S3. Modified gRNAs enhance the CRISPR-dCas13 labeling on RNAs in zebrafish embryos. Fig. S4. Screening the endogenous mRNAs with repeated sequences for labeling. Fig. S5. The labeling capacity of dPspCas13b and dRfxCas13d systems on RNA. Fig. S6. The simultaneous delivery of dCas13 protein and gRNA enhances the labeling capability. Fig. S7. Visualization of other endogenous mRNAs using the CRISPR-dPspCas13b system. Fig. S8. Tracking eppk1 and 100537515 de novo transcription by CRISPR-dPspCas13b. Fig. S9. Post-mitotic transcriptional re-activation of eppk1 and 100537515. Fig. S10. The difference of inter-allele transcriptional activity over time during de novo transcription and post-mitotic re-activation. Fig. S11. The correlation of inter-alleles during de novo transcription and post-mitotic transcriptional re-activation. Fig. S12. Different motion types of 100537515 mRNPs in the nucleus and the cytoplasm. Fig. S13. Different patterns of dPspCas13b/100537515 mRNP export. Fig. S14. Transport factors contribute to dPspCas13b/100537515 mRNP export. [file 13059_2023_2848_MOESM1_ESM.docx]

**Supplementary figures**

**
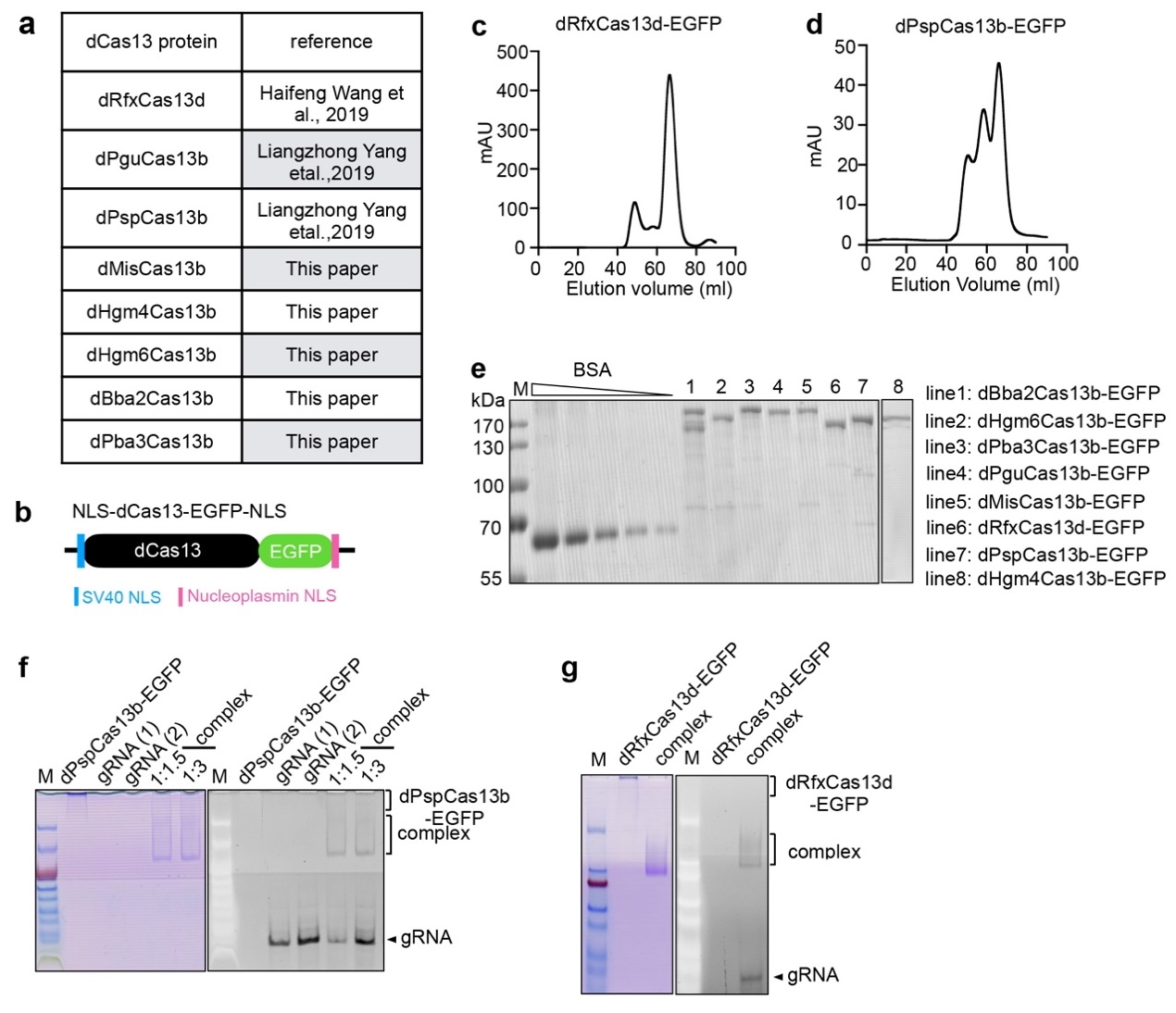
**

**Fig. S1 | dCas13 protein purification and assembly with gRNA**

(a) A summary of screened dCas13 proteins in this study. These include reported dRfxCas13d (16), dPspCas13b and dPguCas13b (17), as well as newly identified dBba2Cas13b, dHgm4Cas13b, dHgm6Cas13b, dMisCas13b and dPba3Cas13b (44).

(b) A schematic view of dCas13-EGFP fused SV40 NLS at the 5′ end and Nucleoplasmin NLS at the 3′ end (abbreviated as dCas13-EGFP). Two NLSs ensure dCas13-EGFP protein localization to the nucleus.

(c)-(d) Graph depicting size exclusion chromatography for dRfxCas13d-EGFP (c) and dPspCas13b-EGFP (d). Measured by UV absorbance (mAU) is shown against the elution volume (mL), and proteins of peak region are collected for further to identify.

(e) SDS-PAGE gel of purified dCas13 proteins is detected by Coomassie Brilliant Blue staining. The serial dilutions of standard BSA protein (2 μg, 1 μg, 0.5 μg, 0.25 μg and 0.125 μg) for standard curve to quantify the concentration of dCas13-EGFP protein and a protein marker is shown on the left.

(f)-(g) Native-PAGE gel detects the assembled dPspCas13b-EGFP/*gGCN4* (f) and dRfxCas13d/*gGCN4* (g) complex *in vitro*. dPspCas13b-EGFP and *gGCN4* shows molar ratio at 1:1.5 and 1:3. dRfxCas13d and *gGCN4* shows molar ratio at 1:1.5. The migration of complex is quicker than dCas13-EGFP alone. The protein is first detected by Coomassie Brilliant Blue staining (left panel), then gRNA is detected by EB staining (right panel).

**
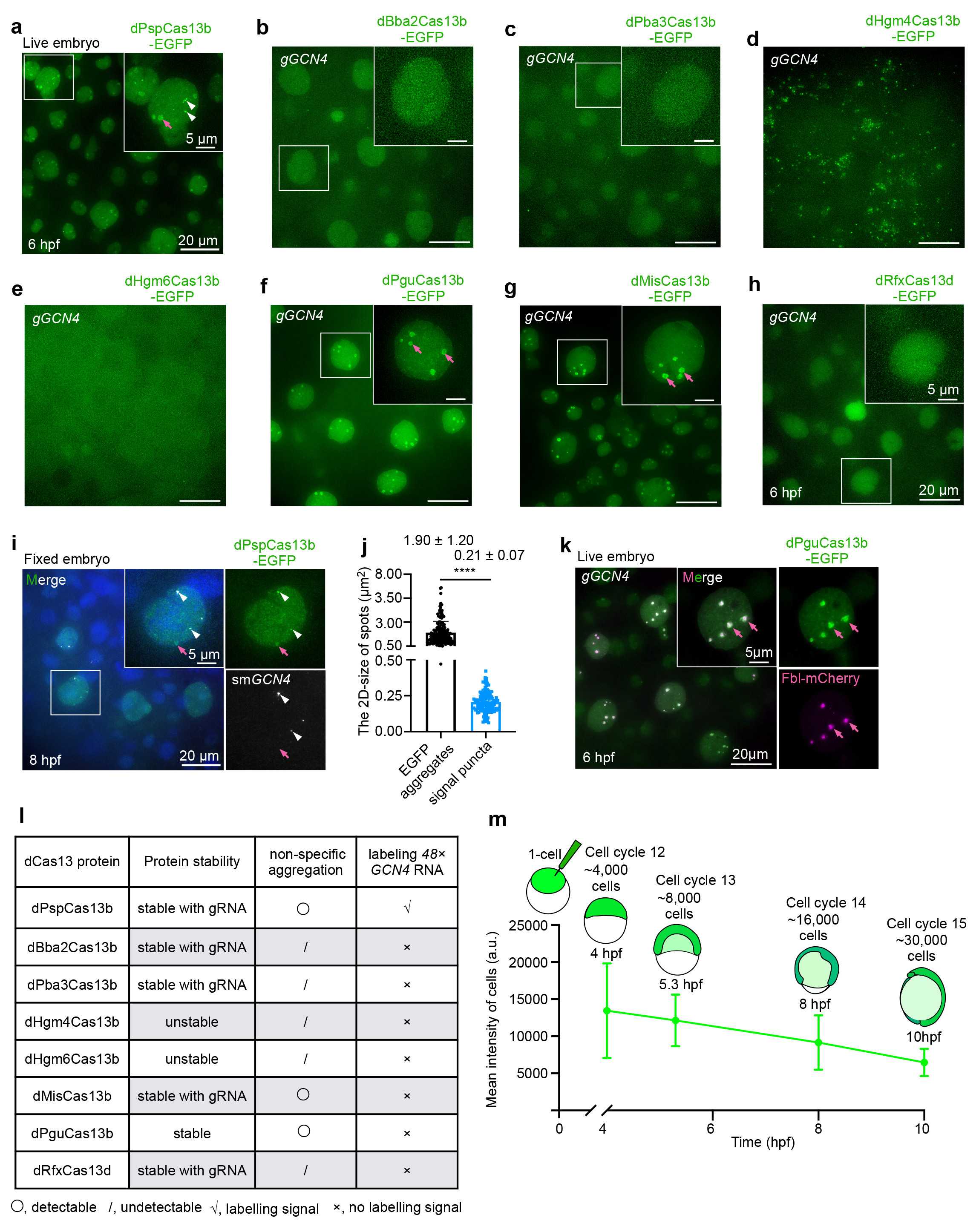
 Fig. S2 | Screening the capable dCas13 proteins to label *48× GCN4* RNA**

(a)-(h) Representative images of CRISPR-dCas13 system labeled *48× GCN4* with no modification *gGCN4* at 6 hpf in live embryos. dPspCas13b (a) has labeling signal in the nucleus as white arrowheads shown. dBba2Cas13b (b), dPba3Cas13b (c) and dRfxCas13d (h) have no labeling signal in the nucleus. In dHgm4Cas13b (d) and dHgm6Cas13b (e), there are undetected obvious EGFP signal in the nucleus which may be attributed to the unstability of dCas13b in zebrafish embryos. EGFP aggregates (magenta arrows shown) are detected upon the injection of dPguCas13b (f) and dMisCas13b (g) complexes, which are also observed upon the injection of dPspCas13b (magenta arrows shown, a).

(i) smFISH confirms dPspCas13b-EGFP targeting *48× GCN4* at 8 hpf in fixed embryos. White arrowheads indicate colocalization between sm*GCN4* FISH signals and dPspCas13b-EGFP puncta in representative embryos. EGFP aggregates are showed by magenta arrows.

(j) The 2D-size of spots between EGFP aggregation and signal puncta. EGFP aggregates are larger than signal puncta. In each group, data from 10 embryos, n= 179, 126 spots; Data are represented as mean ± SD, unpaired two-tail Student’s *t* test, **** *p*< 0.0001.

(k) Representative images of EGFP aggregation localized into nucleolus at 6 hpf. The magenta arrows indicate EGFP aggregates co-localization with Fibrillarin (Fbl)-mCherry.

(l) A summary of screened dCas13 proteins in this study. Examined dCas13 proteins have different properties, including stability, specificity and labeling ability.

(m) A schematic showing that one 1-cell zygotic injection of the proposed amount of CRISPR-dPspCas13b system in (a) enables their distributions to each cell at 10 hpf. The mean intensity of cells is measured by EGFP intensity in the nucleus of each cell from 7-10 embryos at each development time point, including 4 hpf (most cells complete cell cycle 12, about 4,000 cells), 5.3 hpf (most cells complete cell cycle 13, about 8,000 cells), 8 hpf (most cells complete cell cycle 14, about 16,000 cells) and 10 hpf (most cells complete cell cycle 15, about 30,000 cells). Data are represented as mean ± SD.

In (a-c, f-i and k), data scale bar 20 μm; white box indicates magnified area, scale bar 5 μm.

**
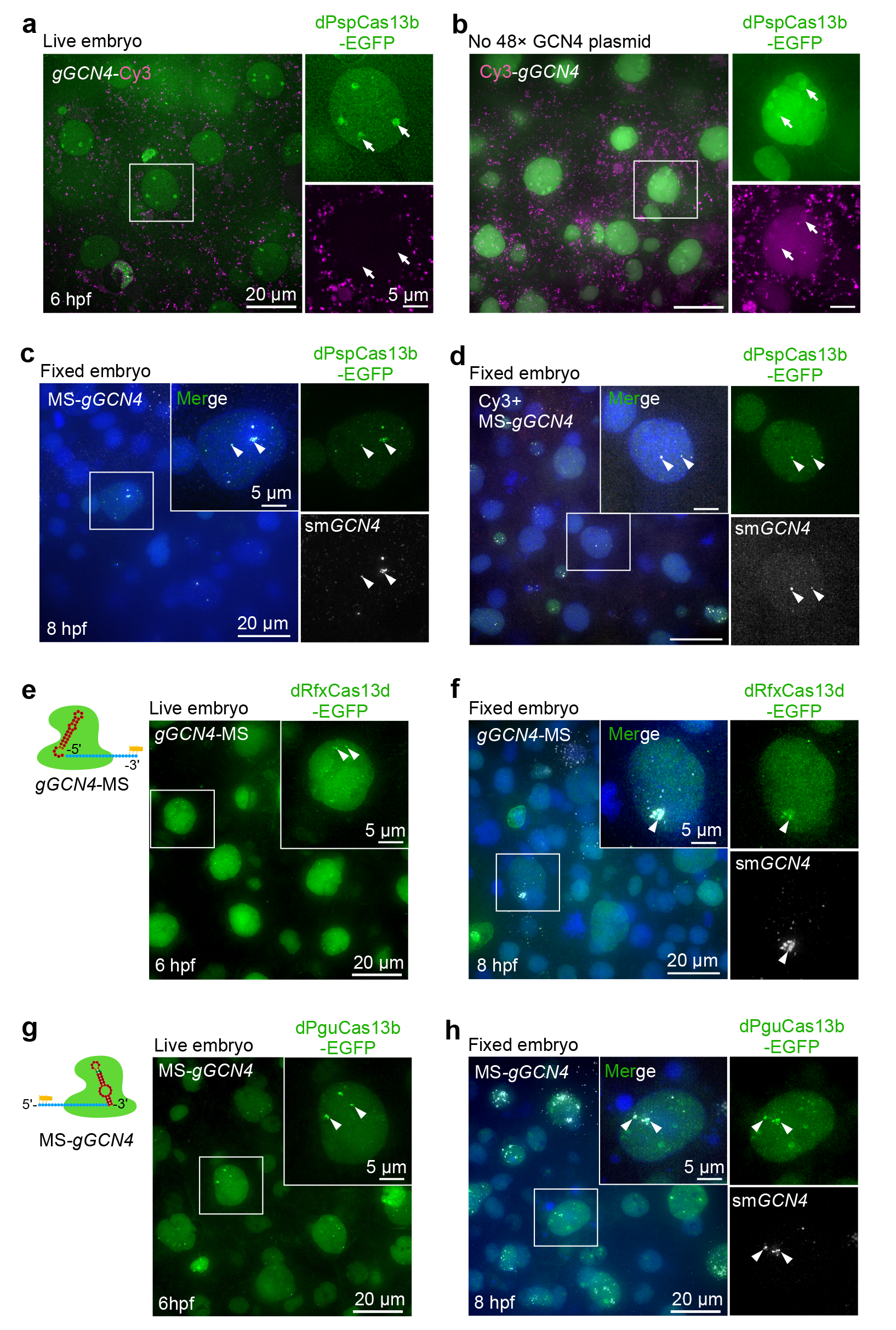
**

**Fig. S3 | Modified gRNAs enhance the CRISPR-dCas13 labeling on RNAs in zebrafish embryos**

(a)-(b) Representative images of CRISPR-dPspCas13b unable to label *48× GCN4* with *gGCN4*-Cy3 (a) and Cy3-*gGCN4* (no injection *48× GCN4* plasmid, b) at 6 hpf in live embryos. White arrows indicate non-specific aggregation in the nucleus of representative embryos.

(c)-(d) smFISH confirms the CRISPR-dPspCas13b system targeting *48× GCN4* with MS-*gGCN4* (c) and Cy3+MS-*gGCN4* (d) at 8 hpf in fixed embryos. White arrowheads indicate colocalization between sm*GCN4* FISH and dPspCas13b-EGFP in representative embryos.

(e) Representative images of CRISPR-dRfxCas13d system-labeled *48× GCN4* with *gGCN4*-MS at 6 hpf in live embryos. Due to spacer of *gGCN4* of dRfxCas13d at 3′ end, 3′ end modified *gGCN4*-MS is applied.

(f) smFISH confirms CRISPR-dRfxCas13d targeting *48× GCN4* at 8 hpf in fixed embryos.

(g) Representative images of CRISPR-dPguCas13b system-labeled *48× GCN4* with MS-*gGCN4* at 6 hpf in live embryos.

(h) smFISH confirms CRISPR-dPguCas13b targeting *48× GCN4* at 8 hpf in fixed embryos.

In (e-h), white arrowheads indicate labeling signals in the nucleus (e and g) and signal colocalization between sm*GCN4* FISH and dCas13-EGFP in the nucleus (f and h). In (a-h), data scale bar 20 μm; white box indicates magnified area, scale bar 5 μm.

**
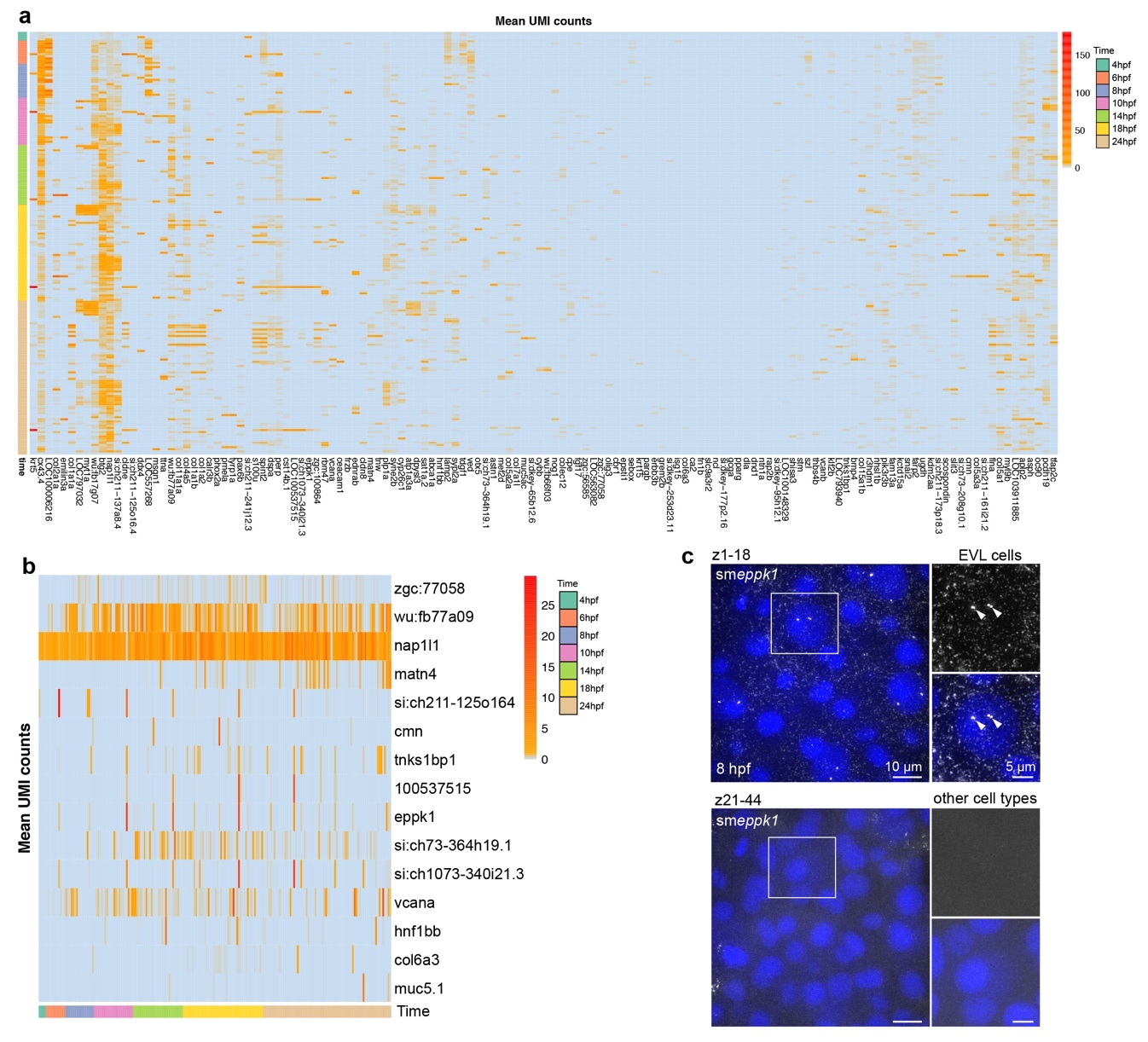
**

**Fig. S4 | Screening the endogenous mRNAs with repeated sequences for labeling**

(a) A pipeline identifies 134 transcripts containing unique repeats and expressed in different cell types from 4 hpf to 24 hpf from sing-cell RNA-seq data. See also Fig. 2a.

(b) Narrowed from (a) to obtain 15 candidate transcripts with at least 8 repeated sequences.

(c) smFISH shows *eppk1* specific expression in EVL cells. sm*eppk1* signals are detected in EVL cells by maximum intensity projection of z-planes 1-18 (Up panel). But sm*eppk1* signals are undetected in other cell types by maximum intensity projection of z-planes 21-44 (down panel). Data scale bar 10 μm. White arrowheads indicate sm*eppk1* signal at the transcription sites. White box indicates magnified area, scale bar 5 μm.

**
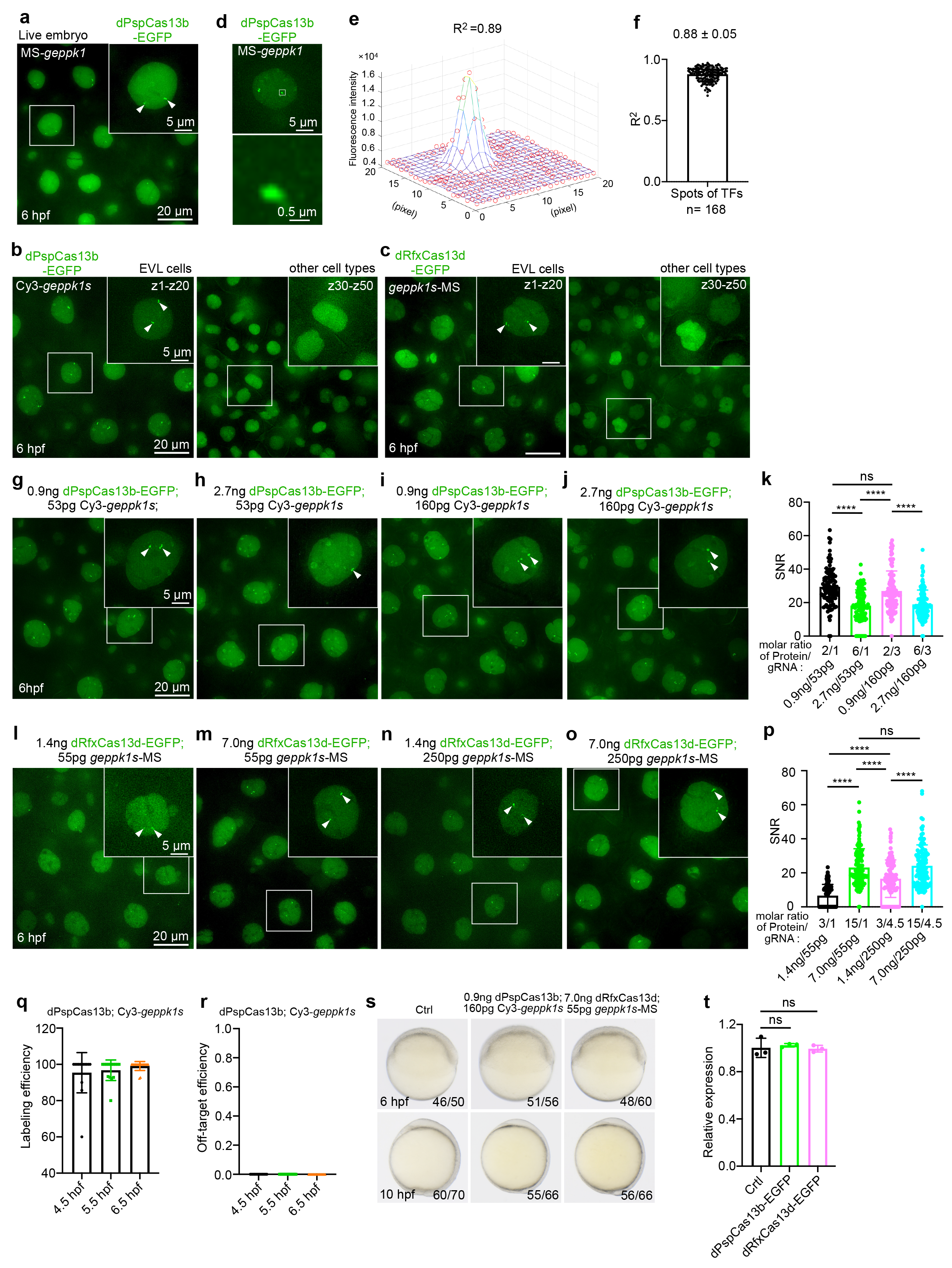
 Fig. S5 | The labeling capacity of dPspCas13b and dRfxCas13d systems on RNA**

(a) Representative images of CRISPR-dPspCas13b system-labeled *eppk1* with individual MS-*geppk1* at 6 hpf in live embryos.

(b)-(c) Representative images of CRISPR-dPspCas13b (b) and -dRfxCas13d (c) systems specifically targeting *eppk1* with three modified gRNAs, Cy3-*geppk1s* and *geppk1s*-MS, respectively, in the EVL cells at 6 hpf in live embryos. Labeling signals detect in EVL cells by maximum intensity projection of z-planes 1-20 (left panel). But sm*eppk1* signals are undetected in other cell types by maximum intensity projection of z-planes 30-40 (right panel).

(d)-(f) The spots of transcription-sites fitted with 2D-Gaussian function. The representative of spots in transcription sites (d), scale bar 5 μm; white box indicates magnified area, scale bar 0.5 μm. The representative of spot (d) fitted with 2D-Gaussian function (e). The mean R^2^ of total 168 spots (f); TFs, transcription sites; mean ± SD.

(g)-(j) Representative images of different concentration of CRISPR-dPspCas13b system-labeled *eppk1* with Cy3-*geppk1s* at 6 hpf in live embryos. Injected each embryo with 0.9 ng dPspCas13b/53 pg Cy3-*geppk1s* (molar ratio 2/1, g), 2.7 ng dPspCas13b/53 pg Cy3-*geppk1s* (molar ratio 6/1, h), 0.9 ng dPspCas13b/160 pg Cy3-*geppk1s* (molar ratio 2/3, i) and 2.7 ng dPspCas13b/160 pg Cy3-*geppk1s* (molar ratio 6/3, j).

(k) SNR statistics of *eppk1* signals labeled by different concentration of CRISPR-dPspCas13b system (g-j). Data from about 10 embryos in each group; n= 108, 112, 107, 112 cells.

(l)-(o) Representative images of different concentration of CRISPR-dRfxCas13d system-labeled *eppk1* with *geppk1s-*MS at 6 hpf in live embryos. Injected each embryo with 1.4 ng dRfxCas13d/ 55 pg *geppk1s*-MS (molar ratio 3/1, l), 7.0 ng dRfxCas13d/55 pg *geppk1s*-MS (molar ratio 15/1, m), 1.4 ng dRfxCas13d/250 pg *geppk1s*-MS (molar ratio 3/4.5, n) and 7.0 ng dRfxCas13d/250 pg *geppk1s*-MS (molar ratio 15/4.5, o).

(p) SNR statistics of *eppk1* signals labeled by different concentration of CRISPR-dRfxCas13d system (l-o). Data from about 10 embryos in each group; n= 130, 118, 101, 111 cells.

(q)-(r) The labeling (q) and off-target (r) efficiency of CRISPR-dPspCas13b system-labeled *eppk1* in transcription sites from 4.5 hpf to 6.5 hpf. 0.9 ng dPspCas13b/160 pg Cy3-*geppk1s* was microinjected. In each group data from n= 14, 15, 17 embryos.

(s) CRISPR-dPspCas13b/-dRfxCas13d system have no obvious toxicity in embryo development at 6 hpf and 10 hpf. Compared to no injection in each embryo as control (Ctrl, left), 0.9 ng dPspCas13b/160 pg Cy3-*geppk1s* (middle) and 7.0 ng dRfxCas13d/55 pg *geppk1s*-MS injection in each embryo show that embryos are developmentally normal. N1/N2, for example 46/50, in all 50 examined embryos, 46 embryos as shown in representative embryos.

(t) QPCR detects that *eppk1* expression has no difference between injection of CRISPR-dPspCas13b/-dRfxCas13d systems and no injection control (Ctrl).

In (a-c, g-j and l-o), data scale bar 20 μm; white arrowheads indicate labeling signals at the transcription sites; white box indicates magnified area, scale bar 5 μm. In (f, k, p-r and t) data are represented as mean ± SD; unpaired two-tail Student’s *t* test; **** *p*< 0.0001; ns, not significant.

**
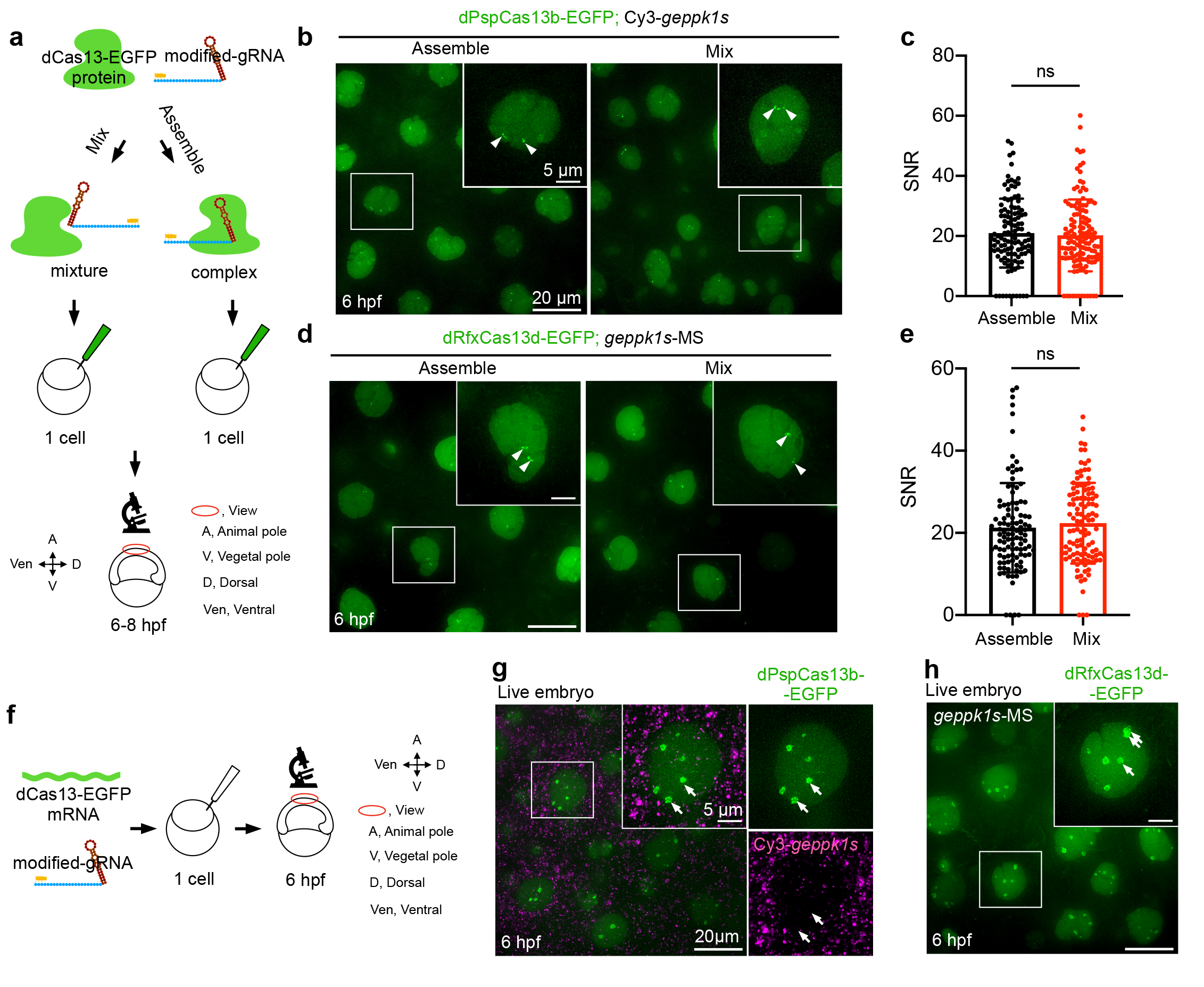
**

**Fig. S6 | The simultaneous delivery of dCas13 protein and gRNA enhances the labeling capability**

(a) A schematic view of injection of assembled dCas13-EGFP/modified-gRNA complex and dCas13-EGFP/modified-gRNA mixture into zebrafish embryos for RNA labeling, respectively.

(b)-(e) Pre-assembled dPspCas13b-EGFP/modified-gRNA complex is not necessary for RNA labeling. Representative images of CRISPR-dPspCas13b (b)/-dRfxCas13d (d) system-labeled *eppk1* with Cy3-*geppk1s* and *geppk1s*-MS respectively between assembled complex and mixture injection at 6 hpf in live embryos, as shown (a). SNR statistics of *eppk1* signals labeled by dPspCa13b-EGFP/Cy3-*geppk1s* (c) and dRfxCas13d-EGFP/*geppk1s*-MS (e) with different strategies of delivery (a) shows no significant difference. Data from about 10 embryos in each group; n= 115, 132 cells (c); n= 108, 113 cells (e).

(f) A schematic view of injection of dCas13b-EGFP mRNA with modified gRNA in zebrafish embryos for RNA labeling.

(g)-(h) Representative images of CRISPR-dPspCas13b (g)/-dRfxCas13d (h) system unable to label *eppk1* at 6 hpf in live embryos using delivery strategy of (f).

In (b and d) data, white arrowheads indicate labeling signals, scale bar 20 μm. In (g and h) data, white arrows indicate non-specific aggregation signals. In (b, d, g and h) data, white box indicates magnified area, scale bar 5 μm. In (c and e) Data are represented as mean ± SD; unpaired two-tail Student’s *t* test; ns, not significant.

**
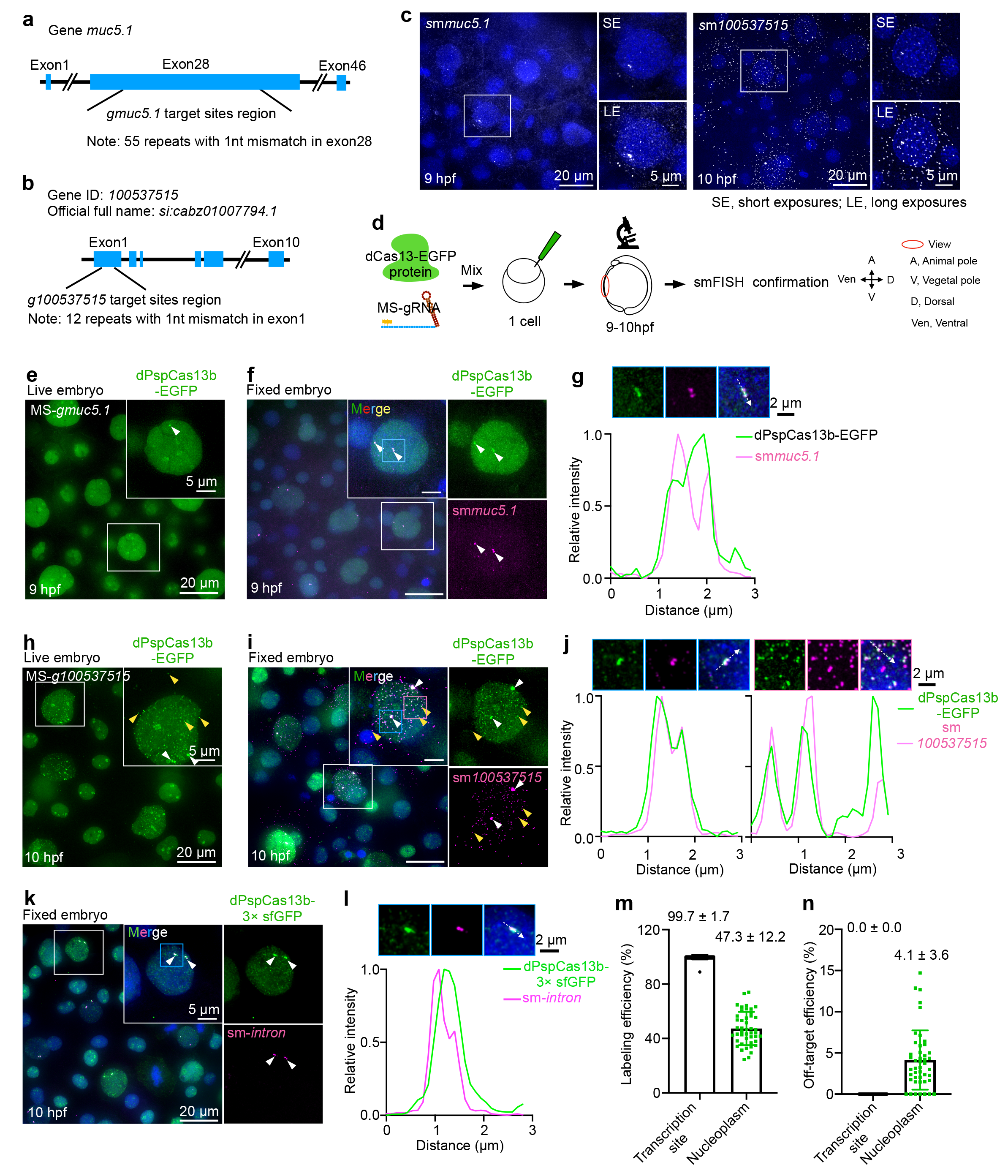
 Fig. S7 | Visualization of endogenous mRNAs using the CRISPR-dPspCas13b system**

(a)-(b) A schematic view of *muc5.1* and *100537515* loci. For *muc5.1* contains repeated sequences in exon 28 (a) and *100537515* locus contains repeated sequences in exon 1 (b). Transcripts of *muc5.1* and *100537515* are predicated to be about 15,900 nt and 2,800 nt, respectively. Designing and screening four MS-*gmuc5.1s* and seven MS-*g100537515s* to target repeated sequences with dPspCas13b-EGFP respectively.

(c) smFISH detects *muc5.1* and *100537515* expression in EVL cells. SE, short exposures, LE, long exposures.

(d) A schematic view of CRISPR-dPspCas13b system to target mRNA in zebrafish embryos. After injecting dPspCas13b protein with MS-gRNA mixture, smFISH confirms labeling signals.

(e) Representative images of CRISPR-dPspCas13b system-labeled *muc5.1* with individual MS-*gmuc5.1* at 9 hpf in live embryos. Four different MS-*gmuc5.1s* are screened.

(f) smFISH confirms dPspCas13b-EGFP/MS-*gmuc5.1* targeting *muc5.1* mRNA at 9 hpf in fixed embryos. Blue box, zoomed in (g).

(g) Analysis of the blue box region in (f). Line scan of the relative fluorescence intensity of signals (white dot line arrow in upper panel) shows the colocalization of dPspCas13b-EGFP and sm*muc5.1* FISH, scale bar 2 μm.

(h) Representative images of CRISPR-dPspCas13b system-labeled *100537515* mRNA with individual MS-*g100537515* at 10 hpf in live embryos. Seven different MS-*g100537515s* are screened. Of note, dPspCas13b-EGFP targets *100537515* mRNA in the nucleus and cytoplasm.

(i) smFISH confirms dPspCas13b-EGFP/MS-*g100537515* targeting *100537515* mRNA in the nucleus and cytoplasm at 10 hpf in fixed embryos. Blue and magenta box, zoomed in (j).

(j) Analysis of the blue and magenta box region in (i). Line scan of the relative fluorescence intensity of signals (white dot line arrows in upper panels) shows the colocalization of dPspCas13b-EGFP and sm*100537515* FISH. It should be noted that a little GFP-only spots were observed, which might be caused by unknown aggregation of dPspCas13b-GFP proteins, or by single molecule probes inefficiently targeting these dPspCas13b/100537515 mRNPs. Scale bar 2 μm.

(k) smFISH confirms dPspCas13b-3*×* sfGFP/MS-*g100537515* targeting intron of nascent *100537515* mRNA in the transcription sites at 10 hpf in fixed embryos. Blue box zoomed in (l).

(l) Analysis of the blue box region in (k). Line scan of the relative fluorescence intensity of signals (white dot line arrow in upper panel) shows the colocalization of dPspCas13b-3*×* sfGFP and sm-*intron* FISH. Scale bar 2 μm.

(m)-(n) The labeling (m) and off-target (n) efficiency of CRISPR-dPspCas13b system-labeled *100537515* in transcription sites and nucleoplasm at 10 hpf. In each group: transcription sites, n= 42 embryos (from 220 cells); nucleoplasm, n= 45 cells (from 15 embryos). Data are represented as mean ± SD.

In (c, e-f, h-i and k), data scale bar 20 μm; white box indicates magnified area, scale bar 5 μm. In (e-f, h-i and k), white arrowheads indicate labeling signals at transcription sites (e-f, h-i and k) and colocalization between dPspCas13b and smFISH (f, i and k); yellow arrowheads indicate labeling signals in the nucleoplasm and cytoplasm (h and i).

**
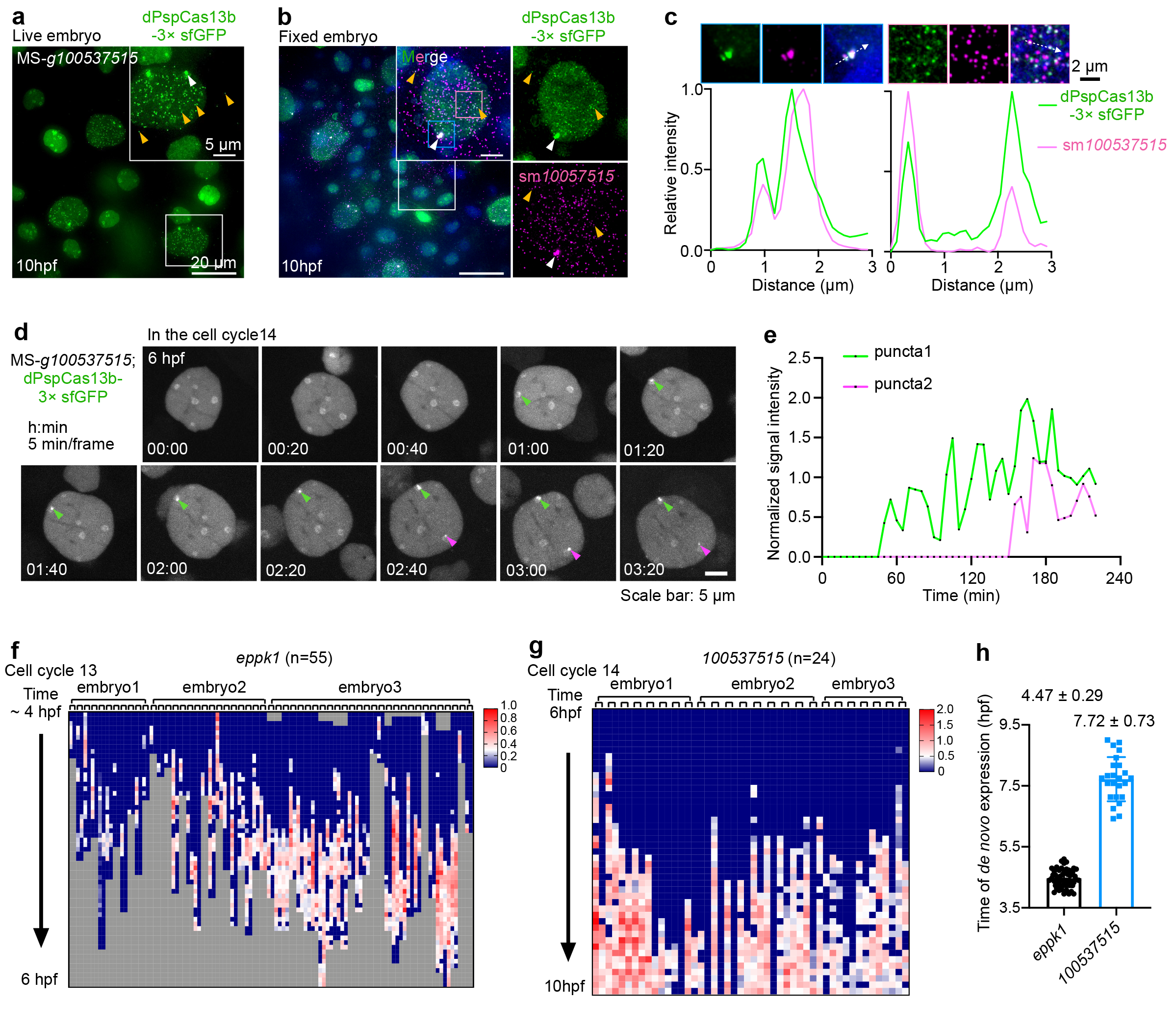
 Fig. S8 | Tracking *eppk1* and *100537515* *de novo* transcription by CRISPR-dPspCas13b**

(a) Representative images of CRISPR-dPspCas13b system-labeled *100537515* mRNA with individual MS-*g100537515* at 10 hpf in live embryos. dPspCas13b-3*×* sfGFP targets *100537515* mRNA in the nucleus and cytoplasm.

(b) smFISH confirms dPspCas13b-3*×* sfGFP/MS-*g100537515* targeting *100537515* mRNA in the nucleus and cytoplasm at 10 hpf in fixed embryos. Blue and magenta box, zoomed in (c).

(c) Analysis of the blue and magenta box region in (b). Line scan of the relative fluorescence intensity of signals (white dot line arrows in upper panels) shows the colocalization of dPspCas13b-3*×* sfGFP and sm*100537515* FISH, scale bar 2 μm.

(d) Representative images of dPspCas13b-3*×* sfGFP tracking *de novo* transcription of *100537515* with MS-*g100537515* recording every five minutes from 6 hpf to 10 hpf during EVL of cell cycle 14, see also Additional file 7: Movie 4. The two transcription sites are shown by green arrowheads first expressional puncta 1, and magenta arrowheads latterly expressional puncta 2, scale bar 5 μm. Puncta 1 with two distinguishable signal spots in a close proximity after 2 hours: 40 min, which may correspond to transcription sites on sister chromatids (20).

(e) Non-synchronized *de novo* transcription of inter-alleles at *100537515* in developing zebrafish embryos. Normalized signal intensity at transcription sites recorded over time in (d).

(f)-(g) Heterogeneous *de novo* transcription of inter-alleles and EVL cells at *eppk1* and *100537515* in developing zebrafish embryos. Heatmap recording normalized signal intensity indicates transcriptional activity of *eppk1* (f) during cell cycle 13 (from 3 embryos, n= 55 cells) and *100537515* (g) during cell cycle 14 (from 3 embryos, n= 24 cells) in EVL cells. The gray regions in the heatmaps shows EVL cells are in mitosis or other cells cycle interphase.

(h) Time of *de novo* transcription of *eppk1* and *100537515* in different cells. Data are represented as mean ± SD.

In (a and b) data, scale bar 20 μm; white box indicates magnified area, scale bar 5 μm.

**
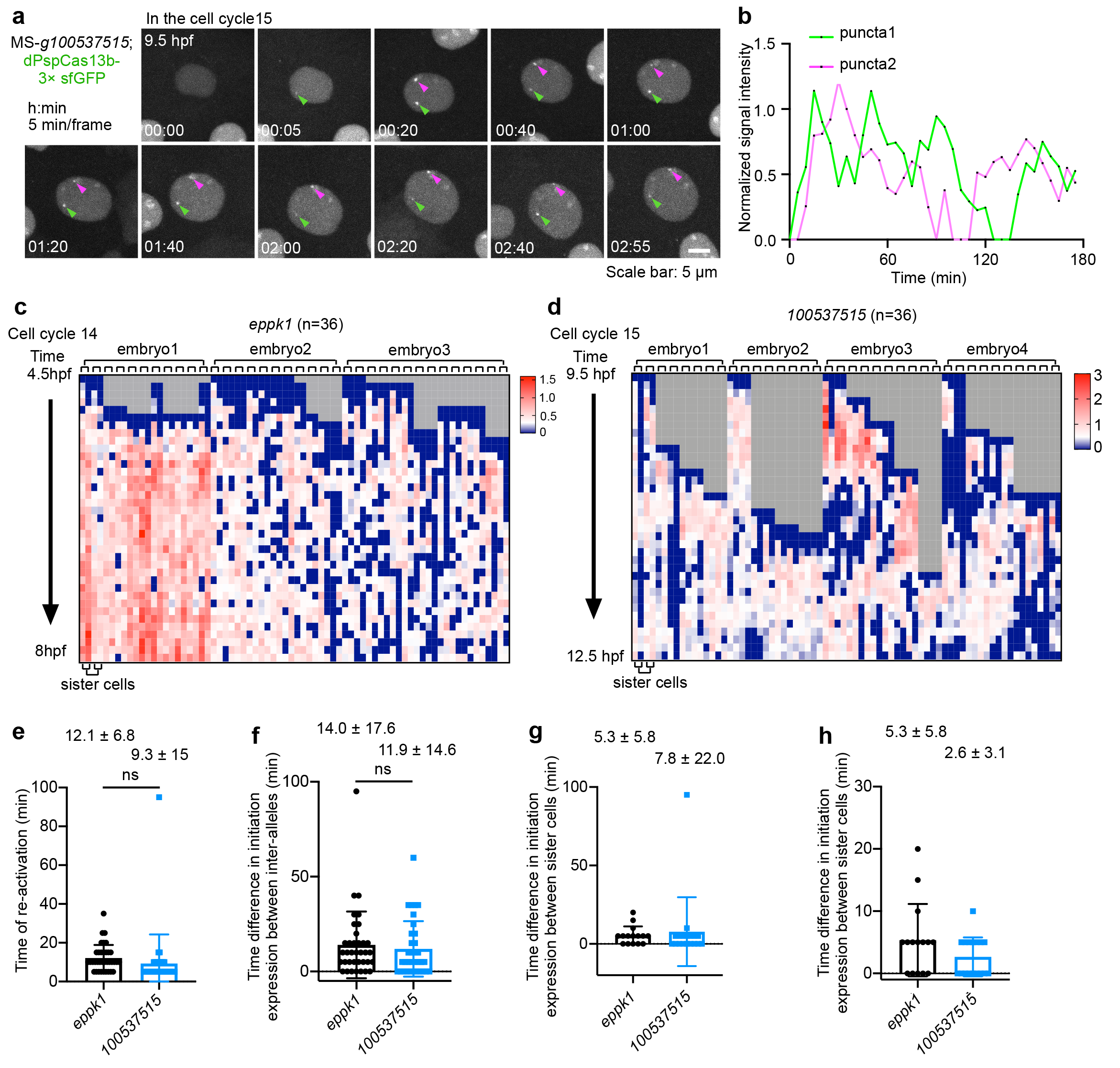
 Fig. S9 | Post-mitotic transcriptional re-activation of *eppk1* and *100537515***

(a) Representative images of dPspCas13b-3*×* sfGFP tracking *100537515* re-activations with MS-*g100537515* recording every five minutes from 9.5 hpf to 13 hpf during EVL of cell cycle 15, see also Additional file 7: Movie 8. Green arrowheads indicate puncta 1, the first detected re-activation transcription site, and magenta arrowheads indicate puncta 2, the other re-activation transcription site in examined cells.

(b) Synchronized transcription of inter-allelic *100537515* in the cell cycle post the cell cycle having *de novo* transcription. Normalized signal intensity at transcription sites recorded over time in (a) in the cell cycle 15 in developing embryos.

(c)-(d) Homogeneous post-mitotic transcriptional re-activation of inter-alleles and EVL cells at *eppk1* and *100537515* in developing zebrafish embryos. Heatmap records transcriptional activity as shown normalized signal intensity of *eppk1* re-activation during in EVL cell cycle 14 (from 3 embryos, n= 36 cells; c) and *100537515* re-activations during EVL cell cycle 15 (from 4 embryos, n= 36 cells; d) in real-time after mitosis. For recording *eppk1* and *100537515* re-activation EVL cells, the mothers of recording daughter cells have been expressed *eppk1* or *100537515*. The gray regions in the heatmaps shows EVL cells are in mitosis or other cells cycle interphase.

(e) Time of transcriptional re-activation between *eppk1* (left) and *100537515* (right) in the next cell cycles (the cell cycle 14 and 15, respectively). Data were traced from Additional file 1: Fig. S9c,d for original data.

(f) Post-mitotic time difference in initial transcription between individual inter-alleles of *eppk1*(left) and *100537515* (right) during transcriptional re-activation in the next cell cycles (the cell cycle 14 and 15, respectively). Data were traced from Additional file 1: Fig. S9c,d for original data. See also Fig. 4d, in which removed one value with the largest error in *eppk1* group.

(g)-(h) Post-mitotic time difference in initial transcription between sister cells of *eppk1*(left) and *100537515* (right) during transcriptional re-activation in the next cell cycles (the cell cycle 14 and 15, respectively). Data were traced from Additional file 1: Fig. S9c,d, *eppk1* for 15 pairs (tracked 21 pairs of *eppk1* sister cells of which 6 sister cells become other type cells), *100537515* for 18 pairs (f). In (g) removed one value with the largest error in *100537515* group. See also Fig. 4e.

In (e-h), data are represented as mean ± SD; ns, not significant.

**
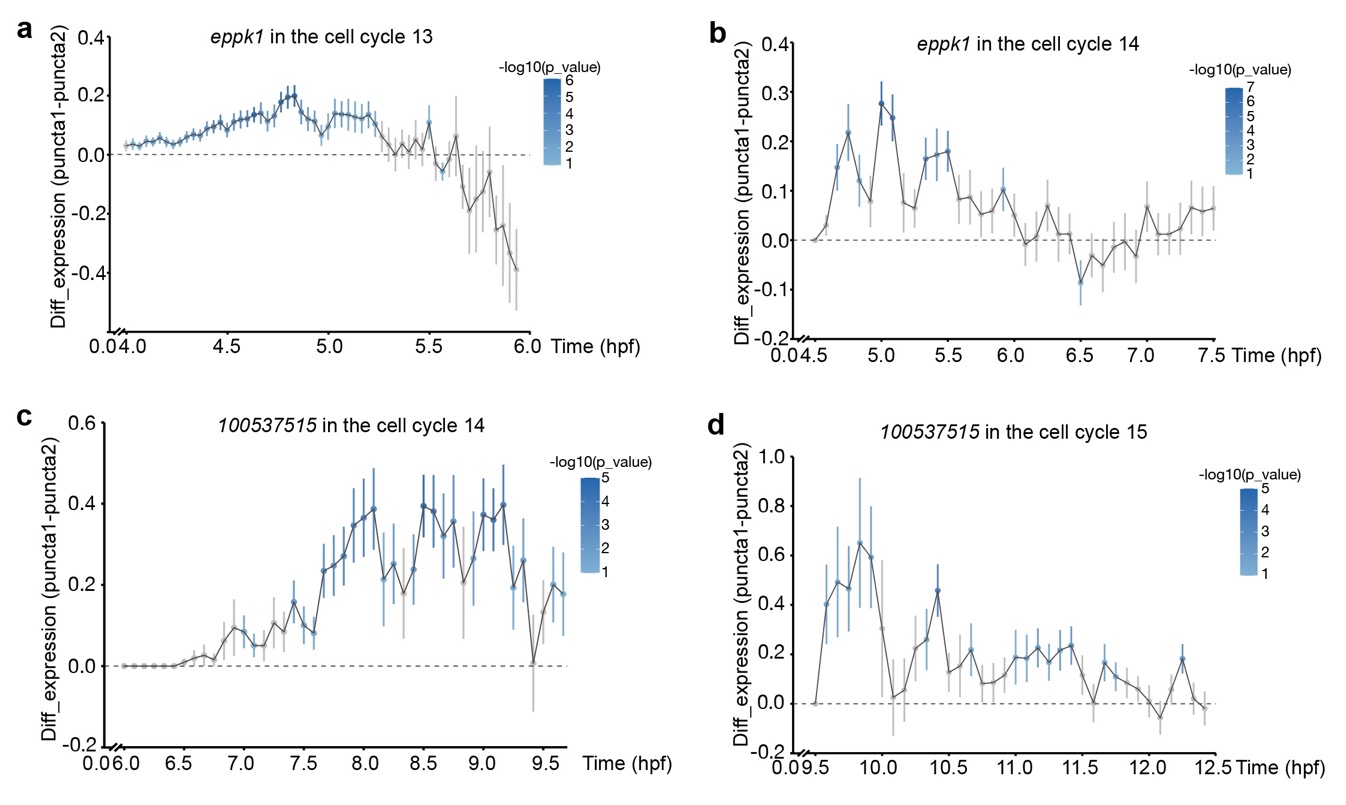
 Fig. S10 | The difference of inter-allele transcriptional activity over time during *de novo* transcription and post-mitotic re-activation**

(a)-(d) Reduction of inter-allelic transcriptional fluctuations from *de novo* transcription to post-mitotic re-activation. Time-dependent curves of differential activity of inter-alleles of all cells by measuring difference (Diff_) expression between puncta 1 and puncta 2 in each cell over time. The difference in transcriptional activity of *eppk1* in EVL of cell cycle 13 (n= 55 cells, a) and *100537515* in EVL of cell cycle 14 (n= 24 cells, c) is gradually increased to peak and have significant difference in inter-allele during *de novo* expression. By contrast difference in transcriptional activity of *eppk1* in EVL of cell cycle 14 (n= 36 cells, b) and *100537515* in EVL of cell cycle 15 (n= 36 cells, d) is rapid increased to peak during re-activation, in which reduces the time of initial transcription fluctuations. Each time point, *p*-values from paired two-tailed Student's *t* test for puncta 1 and puncta 2 in all examined cells are shown a color gradient, and time points with *p*> 0.1 are shown in gray. Traces from Additional file 1: Fig. S8f,g and S9c,d for original data. Data are represented as mean ± SD; paired two-tailed Student's *t* test.S

**
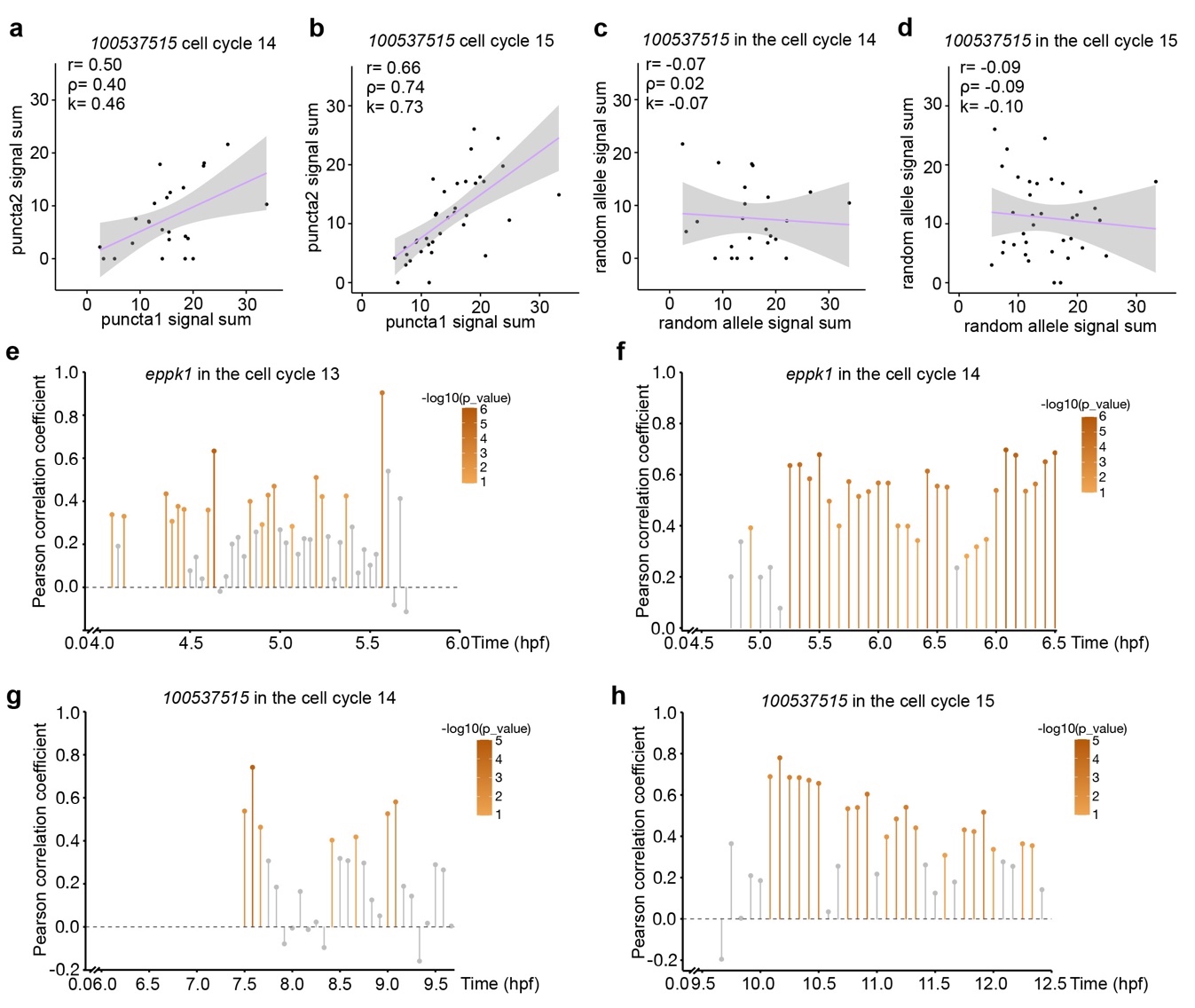
 Fig. S11 | The correlation of inter-alleles during *de novo* transcription and post-mitotic transcriptional re-activation**

(a)-(d) Increased inter-allelic correlations of *100537515* during post-mitotic transcriptional re-activation, compared with those during *de novo* expression. Scatterplot of *100537515* alleles transcriptional output pairs allelic *100537515* in the same cells (a-b) and random pairs allelic*100537515* from the different cells (c-d) during EVL of cell cycle 14 (*de novo* expression, a and c) and cell cycle 15 (post-mitotic transcriptional re-activation, b and d), respectively. Total RNA output is determined by summing the area under the time point traced at each allele (data from Additional file 1: Fig. S8g and S9d). r: Pearson correlation coefficient, ρ: Spearman's rank correlation coefficient, k: Slope. The pink line in each panel indicates slope.

(e)-(h) Inter-allelic positive correlation of *eppk1* in EVL of cell cycle 13 (e) and cell cycle 14 (f), as well as *100537515* in EVL of cell cycle 14 (g) and cell cycle 15 (h) over time. The height of the solid circles in the figure represents the Pearson correlation coefficient of puncta 1 and puncta 2 expression at each time point (the correlation coefficient at each time points quantified cell number >= 5). The *p*-values for the correlation test are shown with a color gradient, and the time points with *p*> 0.1 are shown in gray; unpaired two-tailed Student's *t* test. Traces from Additional file 1: Fig. 8f,g and S9c,d. See also Fig. 4l,m.

**
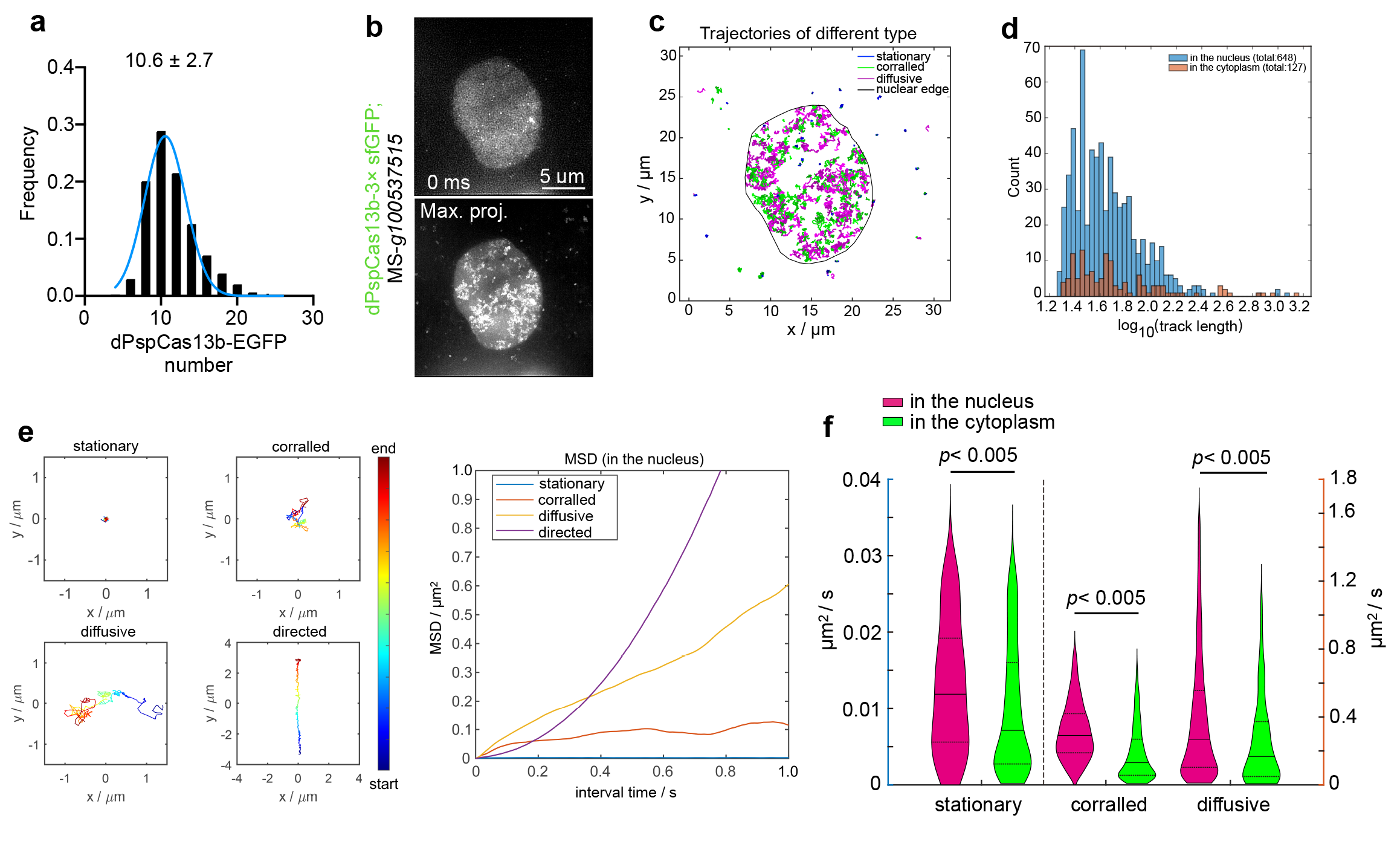
 Fig. S12 | Different motion types of *100537515* mRNPs in the nucleus and the cytoplasm**

(a) Each mRNP contained ~11 copies of dPspCas13b-EGFP. According to standard curve of purified dPspCas13b-EGFP protein at different concentrations, we obtained the distribution of the number of fluorescent molecules for each mRNP. Total 1,833 dPspCas13b-EGFP-engaged *100537515* mRNPs were recorded and fitted with Gaussian function; Data represents as mean ± SD, R^2^ = 0.95.

(b) Representative image of dPspCas13b-3*×* sfGFP-labeled *100537515* targeting mRNPs in the nucleus and cytoplasm (Up panel). Maximum time projection (max. proj.) of the whole dPspCas13b-3*×* sfGFP movie sequence depicting all mRNP movements (down panel).

(c) Representative trajectories of tracked mRNP movements in the nucleus and cytoplasm. Graph shows three types characterized diffusion, including stationary (blue line), corralled (green line) and diffusive (magenta line) movements. Black circle indicates nuclear edge. See also Additional file 1: Fig. S12d,e and Table 1 for details.

(d) Representative distribution of tracked length (>= 20 frames) in the nucleus and cytoplasm, tracked mRNPs from (c).

(e) Representative trajectories (left) and relative mean-square displacement (MSD) versus time (right) of four types characterized diffusion in the nucleus. Stationary motion maximum trajectory distance is less than 400 nm over a large number of frames, the plot of MSD-*t* nearly parallel to the x-coordinate (blue curve). Corralled movement has a larger motion area (yellow curve) compared to stationary motion; the plot of MSD-*t* begins linearly but reaches a plateau. Diffusive movement (red cure) shows no restrictions in the motion area; plot of MSD-*t* is nearly linear. Occasionally, directed motion (purple curve) is detected, which traveled over long distances (> 2 μm); most of them show a linear trajectory. The time of trajectories from start to end with a color gradient. See also Table 1, Additional file 1: Fig. S12f and Additional file 7: Movies 9-12 for details.

(f) mRNPs diffusion in the nucleus faster than in the cytoplasm. Graph depicting shows diffusion coefficient (μm^2^/s) of stationary, corralled and diffusive movements from 17 cells in the nucleus and cytoplasm; see also Table 1 for details. Mann-Whitney test was used. Center line, median; upper and lower lines, 25% and 75% quartiles.

**
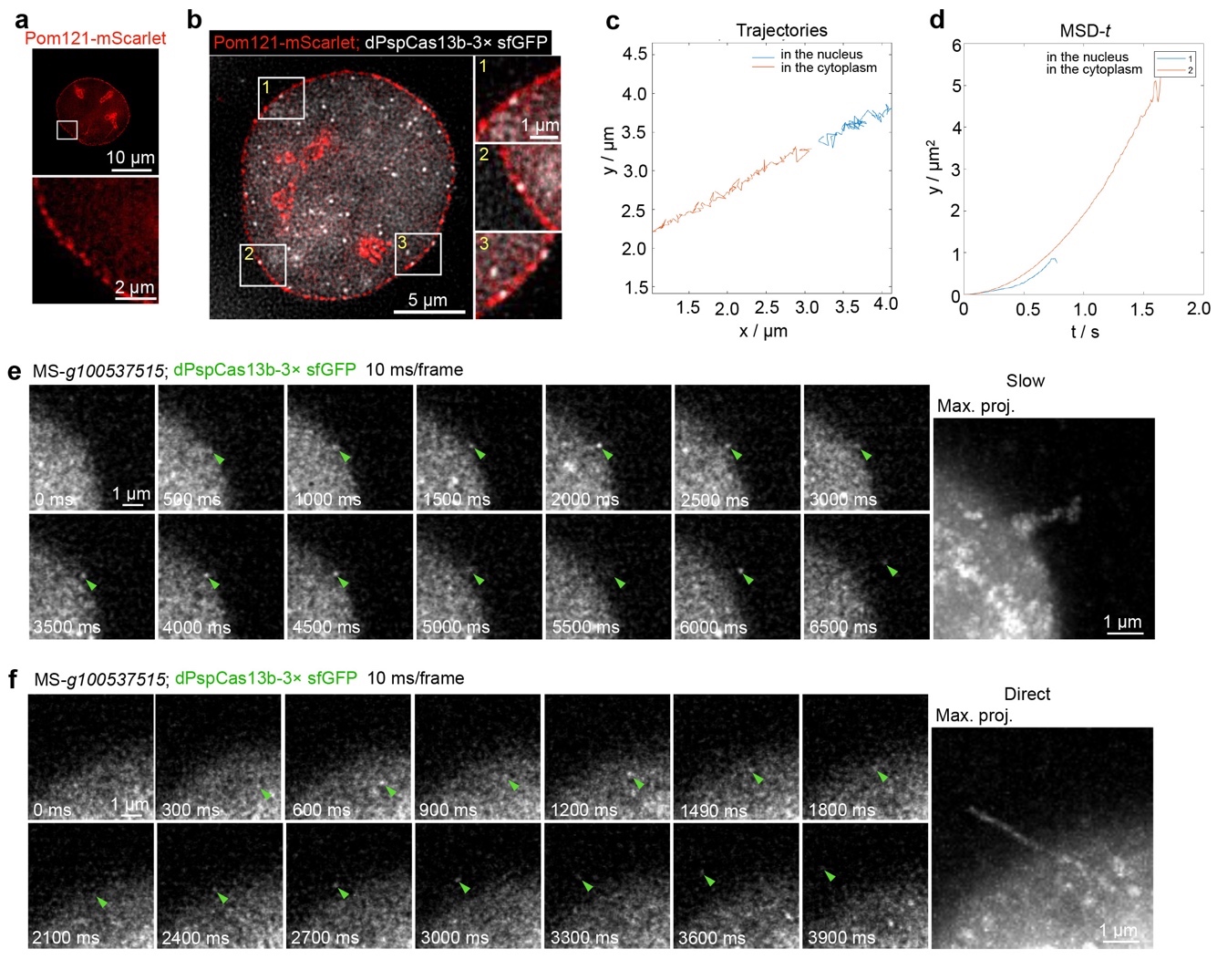
 Fig. S13 | Different patterns of dPspCas13b/*100537515* mRNP export**

(a) Representative images of Pom121-mScarlet marking NPCs. Scale bar 10 μm. White box shows magnified area, scale bar 2 μm.

(b) Representative images of mRNPs dwell on the nuclear pore for a long time. Scale bar 5 μm. White box shows magnified area, scale bar 1 μm. See also Additional file 7: Movie 13.

(c) Representative trajectory of direct export event traveling from the nucleus (blue trajectory) to cytoplasm (yellow trajectory). See also Additional file 1: Fig. S13d,f and Additional file 7: Movie 16.

(d) Mean-square displacement (MSD) versus time shows the comparison of kinetics in the nucleus and the cytoplasm. Graph depicts the same type movement, directed diffusion. See also Additional file 1: Fig. S13c,f; Additional file 7: Movie 16.

(e)-(f) Representative images from the slow export event (e) and direct export event (f) recording every 10 ms. mRNPs in each selected frame is shown by green arrowheads. Maximum time projection (max. proj.,) of movie shows trajectory of mRNPs export, scale bar 1 μm. See also Additional file 7: Movies 15 and 16.

**
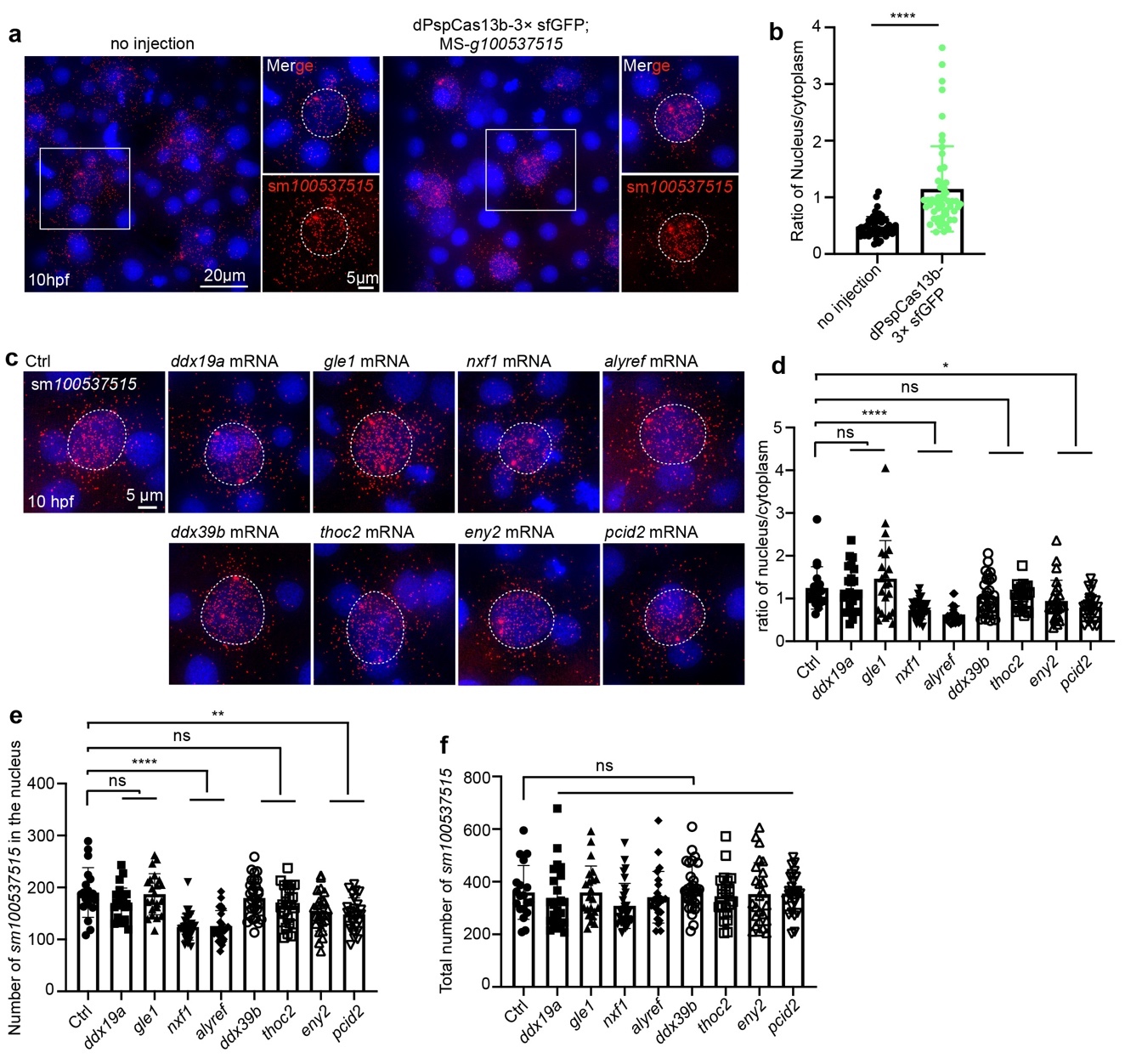
 Fig. S14 | Transport factors contribute to dPspCas13b/*100537515* mRNP export**

(a) Representative images of sm*100537515* signals distribution in the nucleus and cytoplasm between injected CRISPR-dPspCas13b system and no injection control (Ctrl) at 10 hpf. Dot circle indicates nuclear, as examined by DAPI staining, scale bar 20 μm. White box shows magnified area, scale bar 5 μm.

(b) Distribution of *100537515* mRNAs is increased in the nucleus after CRISPR-dPspCas13b system injection. Statistics of nucleus/cytoplasm ratio show that sm*100537515* signals are increased in the nucleus after CRISPR-dPspCas13b system injection (left), compared with no injection (right) at 10 hpf in the data (a).

(c) Representative images of sm*100537515* signals distribution in the nucleus and cytoplasm after injected transport factors mRNA. Overexpression of each transport factors mRNA, *ddx19a*, *gle1*, *alyref*, *nxf1*, *ddx39b*, *thoc2*, *eny2* and *pcid2* with CRISPR-dPspCas13b system, respectively, compares to the control (Ctrl) only CRISPR-dPspCas13b system injection at 10 hpf in fixed embryos. Dot circle indicates nuclear, as examined by DAPI staining. Scale bar 5 μm.

(d)-(f) Overexpression of transport factors recovered the aberrant distribution of *100537515* mRNA after CRISPR-dPspCas13b system injection. Graph depicting nuclear/cytoplasm ratio of *100537515* mRNA (d), the number of *100537515* mRNA in the nucleus (e) and the total number of *100537515* mRNA (f) from (c). Alyref and Nxf1 are most significantly decreased nuclear/cytoplasm ratio and the number of *100537515* mRNA in the nucleus. The total number of *100537515* mRNA in the nucleus and the cytoplasm have no difference in different group, from about 10 embryos, n= 19, 24, 22, 29, 23, 30, 23, 25, 26 cells.

In (b and d-f) data are represented as mean ± SD; unpaired two-tail Student’s *t* test; ns, not significant; * *p* < 0.05, ** *p*< 0.01, **** *p*< 0.0001. Ctrl, control.
