# Supplementary material for: CRISPR-dCas13-tracing reveals transcriptional memory and limited mRNA export in developing zebrafish embryos
Source: Genome Biol. 2023 Jan 19;24:15. doi: 10.1186/s13059-023-02848-6 (PMC9854193; doi:10.1186/s13059-023-02848-6)
Supplement: Supplementary file 7 — Additional file 7. Movies. The recorded dynamic transcription and mRNP motion in this study. [file 13059_2023_2848_MOESM7_ESM.docx]

**Movies**

**Movie 1 – Movie 8:** CRISPR-dPspCas13b system tracks *eppk1* and *100537515* *de novo* expression and post-mitotic transcriptional re-activation in EVL cells of zebrafish embryo performed with Olympus SpinSR confocal microscopy. Time shows hour: minute (h: min); Arrowheads in representative images, green arrowheads indicate puncta 1, and magenta arrowheads indicate puncta 2. Movies play 5 frames/s.

**Movie 1.** dPspCas13b-EGFP/Cy3-*geppk1s* tracks *de novo* transcription of *eppk1* in population of EVL cells during cell cycle 13 recording every 2 min, from about 4 hpf- 6 hpf.

**Movie 2.** dPspCas13b-3*×* sfGFP/MS-*g100537515* tracks *de novo* transcription of *100537515* in population of EVL cells during cell cycle 14 recording every 5 min, from about 6 hpf- 10 hpf.

**Movie 3.** dPspCas13b-EGFP/Cy3-*geppk1s* tracks *de novo* transcription of *eppk1* in single EVL cell during cell cycle 13 recording every 2 min, from about 4 hpf- 6 hpf.

**Movie 4.** dPspCas13b-3*×* sfGFP/MS-*g100537515* tracks *de novo* transcription of *100537515* in single EVL cell during cell cycle 14 recording every 5 min, from about 6 hpf - 10 hpf.

**Movie 5.** dPspCas13b-EGFP/Cy3-*geppk1s* tracks *eppk1* re-activation in population of EVL cells during cell cycle 14 recording every 5 min, from about 4.5 hpf- 8 hpf.

**Movie 6.** dPspCas13b-3*×* sfGFP/MS-*g100537515* tracks *100537515* re-activation in in population of EVL cells during cell cycle 15 recording every 5 min, from about 9.5 hpf- 13 hpf.

**Movie 7.** dPspCas13b-EGFP/Cy3-*geppk1s* tracks *eppk1* re-activation in single EVL cell after cell division (cell cycle 14) recording every 5 min, from about 4.5 hpf- 8 hpf.

**Movie 8.** dPspCas13b-3*×* sfGFP/MS-*g100537515* tracks *100537515* re-activation in single EVL cell after cell division (cell cycle 15) recording every 5 min, from about 9.5 hpf- 13 hpf.

**Movie 9 – Movie 16:** The motions of mRNPs were tracked by CRISPR-dPspCas13b system performed with Multi-SIM. mRNP signals were enhanced by deconvolution recording with 10 ms per frame at 10 hpf to 12 hpf. The dPspCas13b-3*×*sfGFP/MS-*g100537515* system tracked four types of mRNP movements in the nucleus and different types of export events.

**Movie 9.** Stationary motion of a representative mRNP in the nucleus, the red line shows the trajectory. Scar bar 0.5 μm. Movies play 20 frames/s.

**Movie 10.** Corralled motion of a representative mRNP in the nucleus, the red line shows the trajectory. Scar bar 0.5 μm. Movies play 20 frames/s.

**Movie 11.** Diffusive motion of a representative mRNP in the nucleus, the red line shows the trajectory. Scar bar 0.5 μm. Movies play 20 frames/s.

**Movie 12.** Directed motion of a representative mRNP in the nucleus, the red line shows the trajectory. Scar bar 1 μm. Movies play 20 frames/s.

**Movie 13.** dPspCas13b-3*×* sfGFP/MS-*g100537515* tracks mRNPs dwell long time on nuclear pores marketed by Pom121-mScarlet. Movie plays 50 frames/s.

**Movie 14.** A representative fast export event of mRNP. NPCs are marketed by Pom121-mScarlet. Green line shows the trajectory. Movie plays 20 frames/s.

**Movie 15.** A representative slow export event of mRNP. Green line shows the trajectory. Movie plays 100 frames/s.

**Movie 16.** A representative directed export event of mRNP. Red line in the nuclear envelop and yellow line in the cytoplasm shows linear trajectory with directed diffusion. Movie plays 100 frames/s.

**Movie 17.** The export events of mRNPs were tracked by CRISPR-dPspCas13b system performed with Olympus SpinSR confocal microscopy recording 50 ms per frame. A representative fast and slow export event of mRNP. Blue line shows fast export event, green line shows slow export event. Movie plays 100 frames/s
